# MR1 overexpression correlates with poor clinical prognosis in glioma patients

**DOI:** 10.1093/noajnl/vdab034

**Published:** 2021-02-20

**Authors:** Phillip Kubica, Montserrat Lara-Velazquez, Marpe Bam, Seema Siraj, Irene Ong, Peng Liu, Raj Priya, Shahriar Salamat, Randy R Brutkiewicz, Mahua Dey

**Affiliations:** 1 Department of Neurological Surgery, University of Wisconsin School of Medicine and Public Health, Madison, Wisconsin, USA; 2 Carbone Cancer Center, University of Wisconsin School of Medicine and Public Health, Madison, Wisconsin, USA; 3 Department of Microbiology and Immunology, Indiana University School of Medicine, Indianapolis, Indiana, USA; 4 Stark Neurosciences Research Institute, Indiana University School of Medicine, Indianapolis, Indiana, USA; 5 Department of Pathology, University of Wisconsin School of Medicine and Public Health, Madison, Wisconsin, USA

**Keywords:** glioblastoma, glioma, methylation, MR1, survival

## Abstract

**Background:**

Glioblastoma is the most common adult primary brain tumor with near-universal fatality. Major histocompatibility complex (MHC) class I molecules are important mediators of CD8 activation and can be downregulated by cancer cells to escape immune surveillance. MR1 is a nonclassical MHC-I-like molecule responsible for the activation of a subset of T cells. Although high levels of MR1 expression should enhance cancer cell recognition, various tumors demonstrate MR1 overexpression with unknown implications. Here, we study the role of MR1 in glioma.

**Methods:**

Using multi-omics data from the Cancer Genome Atlas (TCGA), we studied *MR1* expression patterns and its impact on survival for various solid tumors. In glioma specifically, we validated *MR1* expression by histology, elucidate transcriptomic profiles of *MR1* high versus low gliomas. To understand *MR1* expression, we analyzed the methylation status of the **MR1** gene and *MR1* gene-related transcription factor (TF) expression.

**Results:**

*MR1* is overexpressed in all grades of glioma and many other solid cancers. However, only in glioma, *MR1* overexpression correlated with poor overall survival and demonstrated global dysregulation of many immune-related genes in an *MR1*-dependent manner. MR1 overexpression correlated with decreased **MR1** gene methylation and upregulation of predicted *MR1* promoter binding TFs, implying *MR1* gene methylation might regulate MR1 expression in glioma.

**Conclusions:**

Our in silico analysis shows that *MR1* expression is a predictor of clinical outcome in glioma patients and is potentially regulated at the epigenetic level, resulting in immune-related genes dysregulation. These findings need to be validated using independent in vitro and in vivo functional studies.

**Key Points**

- *MR1* is an MHC class I-like molecule that is overexpressed in all grades of glioma and correlates with decreased overall survival.
- Higher expression of *MR1* results in significant dysregulation of immune-modulatory pathways such as antigen presentation and T-cell activation.
- In gliomas, *MR1* overexpression is potentially epigenetically regulated through decreased methylation of the *MR1* promoter and upregulation of several predicted transcription factors that bind to the *MR1* promoter in tumor cells.

**Importance of the Study**

Glioma is the most common primary malignant brain tumor, and high-grade glioma is the most aggressive form of brain cancer with a near 100% recurrence rate. MHC-I molecules play a key role in the antitumor immune response. MR1 is an MHC class I-like molecule that activates innate T cells. For the first time, we show that *MR1* expression in glioma is a predictor of clinical outcome. Patients with low *MR1*-expressing gliomas had a longer overall survival compared to those with high *MR1*-expressing gliomas. This association of *MR1* expression and patient survival was found to be glioma-specific and in all grades of glioma, however not seen in other solid cancers. Furthermore, gene and pathway analysis showed significant dysregulation of immune-modulatory pathways in high *MR1*-expressing individuals. Based on our findings, *MR1* expression could be used as a prognostic marker for glioma and potentially a therapeutic target and should be investigated further with independent functional studies and confirmatory cohort.

Glioblastoma (GBM), a grade IV glioma, is the most lethal brain cancer with no cure and a nearly 100% recurrence rate.^1^ Treatment for GBM includes maximal surgical resection, followed by temozolomide, radiation, and tumor treatment fields.^2,3^ Despite treatment, median overall survival (OS) is only 21 months with few patients surviving more than 2 years.^1,2,4^

Several factors contribute to the poor OS of glioma patients including the aggressiveness of tumors, resistance to therapy, and recurrence over time.^2,5^ A hallmark feature of high-grade gliomas is tumor-induced global immunosuppression.^6^ Additionally, gliomas develop several immune escape mechanisms, many of which target major histocompatibility complex (MHC) class I and class I-like molecules.^7,8^ Because of such strong interactions between glioma and the immune system, several immunotherapeutic strategies are being investigated in the setting of gliomas with varying degrees of success.^9^ MHC-I and class I-like molecules are of particular interest for an immunotherapeutic approach, as antitumor cytotoxic CD8+ T cells recognize various tumor antigens in the context of MHC-I molecules. Consequently, cancer cells downregulate MHC-I expression as a means of immune evasion.^10^

MR1 is a non-polymorphic MHC class I-like molecule encoded in human chromosome 1 with many similarities to canonical class I molecules, but with the added distinction of being located mainly in the endoplasmic reticulum and endosomal vesicles.^11,12^ MR1 presents microbial-derived vitamin B metabolites to an innate T cell called mucosal-associated invariant T cells^13,14^ resulting in their proliferation and secretion of pro-inflammatory cytokines to control infection.^3^ However, the exact involvement of the MR1 in cancer immunology is unknown.

Although expression of MR1 has been demonstrated in a variety of human cancers, there have been no studies analyzing the role of MR1 in glioma. In the present work, we investigated the expression patterns of *MR1* in all grades of glioma and its impact on patient OS. To understand the mechanism of *MR1* expression in glioma, we studied the DNA methylation pattern of promotor regions of the *MR1* gene. Using in silico analysis, we identified transcription factors (TFs) with potential *MR1* binding sites and found that they are differentially expressed in *MR1* high- versus low-expressing gliomas. To understand the effect of *MR1* overexpression in glioma, we analyzed the transcriptomic profile of MR1 high- versus *MR1* low-expressing gliomas. Using histology, we validated MR1 expression at the protein level in grade IV primary versus recurrent glioma and correlated MR1 expression with immune cell infiltration. Finally, we validated expression levels of TFs that might regulate *MR1* expression using qPCR in glioma samples.

## Materials and Methods

### Overview of Sample and Data Collection

An overview of our sample and data collection is included in Supplementary Information and Supplementary Figure 1.

### MR1 Expression

Genomic and clinical data were obtained from The Cancer Genome Atlas (TCGA) (https://portal.gdc.cancer.gov). Patient sample stratification cut points were determined based on maximally selected rank statistics. RNA-seq differential analysis was performed by the Bioconductor package *edgeR* and gene set enrichment analysis (GSEA) was performed by the Bioconductor package *fgsea*. GENT2 software was used to identify solid tumors that had high *MR1* expression compared to other solid tumors.^15^ Here “glioma” refers to combined grades II, III, and IV. Grade IV is also referred to as GBM. A cut point for high versus low *MR1* expression was determined through UCSC Xena Browser and graphed in Prism 8. Primary and recurrent GBM *MR1* expression was found using GlioVis. mRNA expression is shown as the fold change.

### Survival Curves

Glioma survival was analyzed by *MR1* expression with cut point identified by maximally selected rank statistics. Breast, renal, thyroid, melanoma, stomach, and lung cancers were analyzed separately. IDH status and *MR1* expression in glioma survival were also analyzed. Data collected from the UCSC Xena Browser were filtered for either normal tissue or primary tumors. All null samples were removed to keep data relevant. Results were graphed and analyzed with a Mantel–Cox log-rank test in Prism 8.

### Immunohistochemistry

De-identified human sample slides were obtained from Indiana University under institutional IRB approval. Nineteen matched primary and recurrent GBM tumor samples were obtained and deparaffinized in xylene, then rehydrated in ethanol. After antigen retrieval, slides were stained with anti-human MR1 (Abcam) or CD45RO (BD Pharmingen) antibodies. Briefly, slides were blocked with 10% rabbit serum with 1% bovine serum albumin (BSA) in tris-buffered saline (TBS) for 1 h and incubated with primary antibody overnight (MR1 antibody 1:200, CD45RO 1:2000) at 4°C. The next day, a secondary antibody (Abcam, 1:2000) was applied for 1 h at room temperature. The slides were dehydrated, mounted using Mounting Media (Vector Laboratories), and covered with coverslips. The expression level of MR1 and CD45 was graded as follows: for MR1 staining was graded as 0 = no signal detected, + = 33% positive tumor cells, ++ = 66% positive tumor cells, and +++ = more than 66% positive tumor cells. For CD45 tissues were divided in 4 different quadrants and classified according to the number of quadrants with positive cells, 0 quadrants = 0, 1 quadrant = +, 2 quadrants = ++, and more than 2 quadrants = +++.

### Gene Expression and Pathway Analysis

Glioma patients previously identified were used in this analysis and stratified by the *MR1* expression level. A gene’s Z-score was calculated on its log_10_ (FPKMUQ + 1) value across patients with the same grade of glioma. An expressed gene was required to have at least 10 reads in 70% of samples in each group and at least 15 reads across all the samples. Next, gene expression fold changes and associated adjusted *P* values between primary tumor samples were graphed in a volcano plot. Differentially expressed genes (DEGs) were defined as having │log_2_(fold change)│ ≥ 1 and adjusted *P* < .05. DEGs were colored in red. GSEA of Kyoto Encyclopedia of Genes and Genomes (KEGG) and Gene Ontology (GO) gene sets were performed for grade II, III, and IV gliomas stratified by *MR1* expression levels. A gene set with an adjusted *P* < .05 can be considered as significantly enriched for genes with large expression changes between tumor samples of high and low *MR1* expression. Genes with symbols mapped to other genes were removed to avoid ambiguity. GSEA enrichment plots were also generated.

### Methylation Analysis

Known CpG islands associated with *MR1* were isolated from GEO. These were cross-referenced with methylation data from TCGA Wander and expression data from GlioVis to isolate samples containing both metrics. *MR1* expression levels and IDH status were determined in the UCSC Xena Browser for TCGA-LGG/GBM. In each panel, high and low *MR1* expression were graphed to visualize sample distribution. CpG island sample-matched methylation values were plotted with Prism 8 and analyzed with one-way ANOVA for significance. Methylation values have been multiplied by 100 for ease of interpretation while the transcription start site was obtained for reference in TCGA Wanderer.

### TF Identification

A list of 26 putative TFs obtained through MEME and TOMTOM were cross-referenced with GlioVis to identify 4 final candidates that were differentially expressed in *MR1* high versus low gliomas.^16^ Expression levels were found in GlioVis using the TCGA-LGG/GBM dataset and samples were stratified into *MR1* high or *MR1* low for each grade. A one-way ANOVA with Tukey’s post hoc was performed to determine significance. Immunohistochemistry (IHC) samples for TFs were obtained from The Human Protein Atlas (THPA) and plotted by staining level.

### Quantitative PCR

qPCR was performed for each of the above-identified TFs in grade II, III, and IV gliomas. RNA was isolated from 5 grade II, grade III, and grade IV glioma patient samples. cDNA was synthesized from the isolated RNA (Biorad’s iScript). After cDNA was synthesized, qRT-PCR master mix was prepared (Biorad’s SYBR Green) and mixed with primers (Supplementary Table 1).

## Results

### *MR1* Is Differentially Expressed in Many Common Solid Cancers

**Figure 1.**
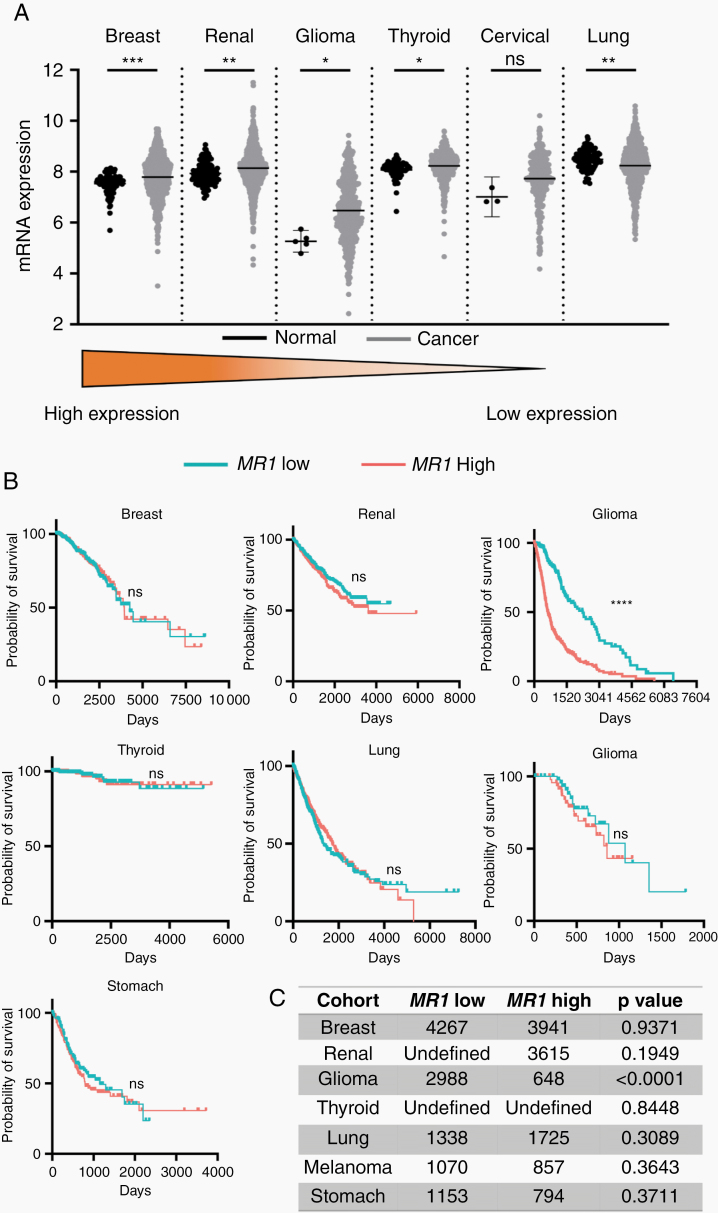
*MR1* is differentially expressed in many common solid cancers. (A) Dot plots comparing *MR1* expression in normal tissue versus primary tumor. (B) Kaplan–Meir curves of various solid cancers with differential *MR1* expression levels show no significant differences in overall survival except for glioma. (C) Median survival (in days) for patients with different solid malignancies stratified by *MR1* expression levels *P < .05, **P < .01, ***P < .0001; ns, not significant.

We analyzed the differential expression of *MR1* in several solid cancers (Figure 1A) in comparison with their normal tissues. Lung cancer has a significantly lower expression of *MR1* compared to normal lung tissue (*P* = .0025). In contrast, breast cancer (*P* < .001), renal cancer (*P* < .0048), glioma (*P* < .0234), and thyroid cancer (*P* < .0395) have significantly higher expression of MR1 compared to their respective normal tissue. *MR1* expression for cervical cancer was not significant in cancerous or non-cancerous tissue (*P* = .2054).

We then compared *MR1* expression levels and patient OS in several common solid cancers including glioma. We saw no statistically significant difference in OS between *MR1* low- versus high-expressing patients, in breast (*P* = .9371), renal (*P* = .1949), thyroid (*P* = .8448), lung (*P* = .3089), melanoma (*P* = .3643), and stomach (*P* = .3711) cancers. However, when analyzing all gliomas together, we saw a statistically significant negative correlation between survival and *MR1* expression (*P* ≤ .0001). This implies that although *MR1* is differentially expressed in many solid cancers, the correlation between MR1 expression and OS is glioma-specific (Figure 1B and C).

### *MR1* Overexpression Is Associated With Worse OS in All Grades of Glioma

**Figure 2.**
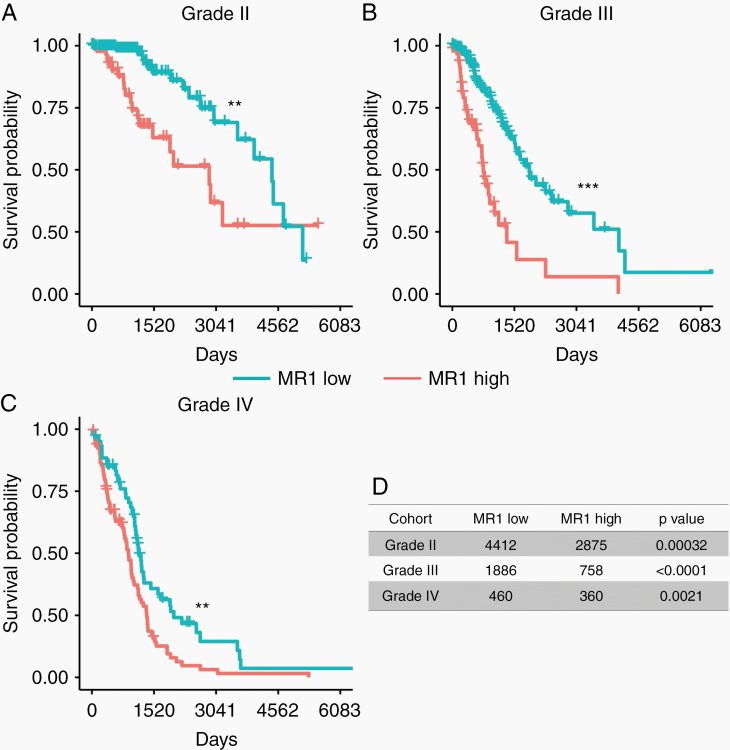
*MR1* overexpression is associated with worse OS in all grades of glioma. (A–C) High *MR1* expression is linked with decreased overall survival in low (II) and high (III, IV) grade gliomas. The cut point was identified through maximally selected rank statistics. (D) Median survival (in days) for glioma patients according to *MR1* expression levels. *P < .05, **P < .01, ***P < .0001; ns, not significant.

To understand the effect of glioma *MR1* expression on patient clinical outcome, we analyzed MR1 expression with the patient’s OS data in all grades of glioma. We stratified patient samples according to cut points that were determined based on maximally selected rank statistics (cut point expression of 50 391, 68 602, and 77 501 for grade II, III, and IV gliomas, respectively; Supplementary Figure 2). Median survival in days for glioma stratified by *MR1* expression showed a statistically significant difference in OS between *MR1* high- versus low-expressing tumors (grade II: *P* < .00032, grade III: *P* < .0001, and grade IV: *P* < .0021; Figure 2A–D). Using Cox proportional hazard ratios test, we found that in GBM patients, poor overall prognosis with high *MR1* expression was irrespective of confounding factors such as age, gender, *MGMT* promoter methylation status, and IDH mutation status (Supplementary Table 2, HR: 1.308, *P* < .004). By analyzing TCGA data, we confirmed that IDH status does not impact the effect of *MR1* on OS. IDH mutation and *MR1* expression are independent factors affecting the clinical outcome of glioma patients (Supplementary Figure 3). Thus, we conclude that *MR1* is an independent prognostic factor, where overexpression is associated with poor OS for patients with all grades of glioma.

### *MR1* Expression Does Not Vary Between Primary and Recurrent GBM

**Figure 3.**
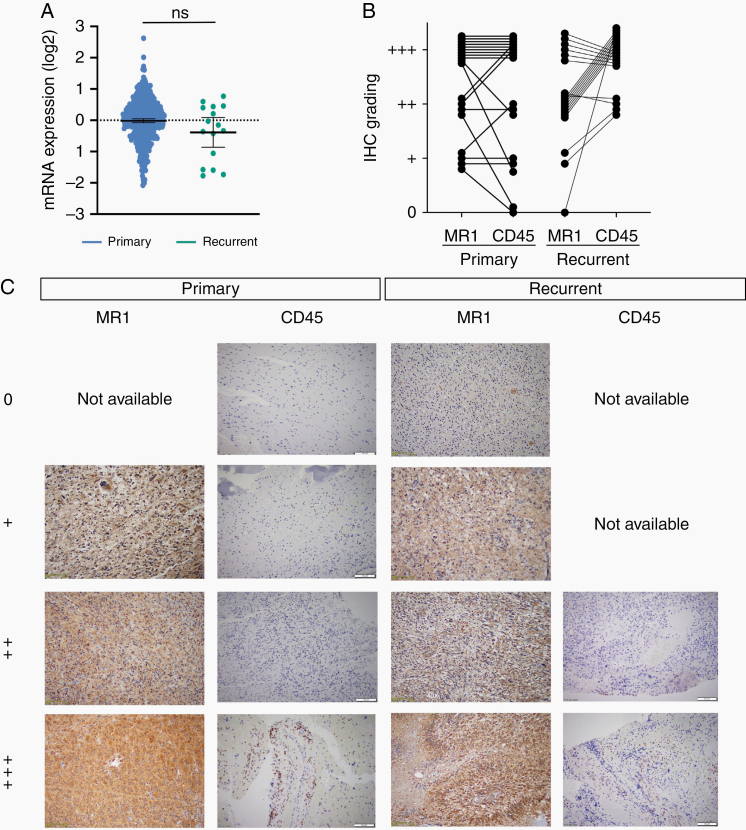
MR1 and CD45 expression does not vary between primary and recurrent glioblastoma. (A) TCGA data show no significant difference between primary and recurrent tumors *MR1* mRNA expression levels in GBM. (B) Quantification of MR1 and CD45 IHC staining levels in matched primary and recurrent GBM tissues. (C) IHC of primary and recurrent tumors that were stained for MR1 and CD45 expression. Staining levels for MR1: 0, not detected; +, low (33%); ++, medium (66%); +++, high (˃66%). For CD45 tissue was divided into 4 quadrants: 0 non-detected, +: 1 quadrant positive, ++: 2 quadrants positive, and +++: ˃2 quadrants positive.

Because grade IV glioma (GBM) is the most common adult glioma and is an incurable disease, associated with 100% recurrence, we analyzed *MR1* mRNA expression levels in primary and recurrent GBM tumors from the TCGA via GlioVis. We found no statistically significant difference in *MR1* expression levels at the RNA level in primary versus recurrent tumors (mean expression of −0.015 vs −0.38, respectively; Figure 3A). In addition, to validate MR1 expression at the protein level, we performed IHC on 19 matched primary and recurrent GBM patient samples (Figure 3B and C). We observed different levels of MR1 staining of the tumor cells in primary versus recurrent GBM, with 0 versus 1 patients showing no (−), 4 versus 2 patients showing low (+), 4 versus 10 patients showing medium (++), and 11 versus 6 showing high (+++) staining. Similar to our finding at the mRNA level, IHC data also confirmed that there was no statistically significant difference in MR1 expression between primary and recurrent GBM, implying that MR1 expression does not increase with tumor recurrence. We also saw that MR1 expression was primarily restricted to the cytoplasm of GBM cells (Supplementary Figure 4). To study if MR1 expression was linked with the recruitment of inflammatory cells in the brain, we evaluated CD45+ cells in the same 19 matched primary and recurrent GBM samples (Figure 3B and C). We observed different levels of CD45 staining in primary versus recurrent tumor patients, with 2 versus 0 patients showing no (−), 3 versus 0 patients showing low (+), 3 versus 4 medium (++), and 11 versus 16 high (+++) staining. Although there is no difference in MR1 expression in primary versus recurrence, recurrent GBM has higher immune cell infiltration compared to primary and mostly MR1 expression level is associated with a higher number of immune cell infiltration. This observation needs further validation using animal models.

### Transcriptomic Signature Varies Between *MR1* Low Versus *MR1* High Gliomas of All Grades

**Figure 4.**
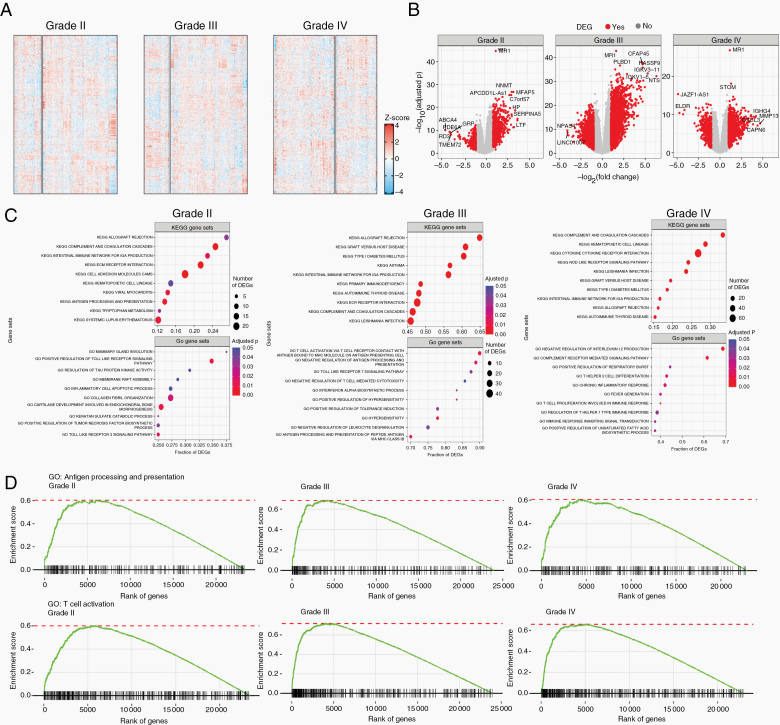
Transcriptomic signature varies between *MR1* low versus *MR1* high gliomas in all grades. (A) Gene expression in 657 glioma patients stratified by *MR1* expression level (high vs low; 246 grade II [66 vs 180], 260 grade III [64 vs 196], and 151 grade IV [90 vs 61]). 70 500 genes were identified (23 485 in grade II, 24 053 in grade III, and 22 962 in grade IV). (B) Gene expression fold changes and associated adjusted *P* values between primary tumor samples stratified by *MR1* expression level. (C) Top 10 KEGG and GO gene sets ranked by the fraction of DEGs for grade II, III, and IV gliomas. Only significantly enriched gene sets (adjusted *P* < .05 were considered). (D) GSEA enrichment plots for selected GO terms.

To understand the transcriptomic landscape of *MR1* low versus high gliomas, we looked at global gene expression levels in these 2 groups based on glioma grade. Transcriptomic heatmaps of 657 gliomas (246 grade II, 260 grade III, and 151 grade IV) detected multiple clusters of dysregulation across glioma grades. For *MR1* high- versus low-expressing gliomas, grade II had 66 versus 180 samples with 23 485 genes differentially expressed; grade III had 64 versus 196 samples with 24 053 genes differentially expressed; and grade IV had 90 versus 61 samples with 22 962 genes differentially expressed (Figure 4A).

To better visualize the genomic landscapes, DEGs were identified and graphed in a volcano plot. An activated DEG is a gene that has higher expression levels in *MR1* high versus low samples. A repressed DEG is a gene that has a lower expression in such comparison. In grade II, the total number of activated versus repressed DEGs were 729 and 459, respectively; for grade III, 2247 versus 1189; and for grade IV, 1051 versus 479. High DEGs in grade II gliomas included NNMT, MFAP5, APCDD1L-AS1, C7orf57, HP, SERPINA5, and LTF and some highly downregulated DEGs included ABCA4, PDE6A, GRP, RD3, and TMEM72. In glioma grade III, highly activated DEGs included CFAP45, PLBD1, RASSF9, IGKV3-11, IGKV1-5, and NTS, while highly downregulated DEGs included NPAS4 and LINC01007. In glioma grade IV, highly activated DEGs included STOM, IGHG4, CXCL13, MMP13, and CAPN6, while highly downregulated DEGs included JAZF1-AS1 and ELDR (Figure 4B). Interestingly, we did not see overlap in the pattern of up- or downregulated genes between groups. Also, no genes were shared between groups, suggesting the effect of *MR1* overexpression on the overall gene expression landscape of glioma is different in different grade implying that aggressiveness of the tumor cell may regulate the effect of *MR1* overexpression on the overall transcriptional landscape of glioma.

Next, to determine the biological processes altered by these dysregulated DEGs in MR1 high versus low tumors, we used KEGG and GO pathway analyses (Figure 4C). In grade II *MR1* high glioma, GO pathways, such as “positive regulation of toll-like receptor signaling, inflammatory cell apoptotic process, positive regulation of tumor necrosis factor biosynthetic process, and toll-like receptor 3 signaling pathways,” and KEGG pathways, such as “antigen processing and presentation, and systemic lupus erythematosus” were dysregulated. In grade III *MR1* high gliomas, GO pathways, such as “T-cell activation via cell receptor contact with antigen bound to MHC molecule on antigen-presenting cell, negative regulation of antigen processing and presentation, toll-like receptor 7 signaling pathway, and negative regulation of T-cell-mediated cytotoxicity, interferon-alpha biosynthetic process, positive regulation of hypersensitivity, positive regulation of tolerance induction, hypersensitivity, negative regulation of leukocyte degranulation, and antigen processing and presentation of peptide antigen via MHC class IB” and KEGG pathways, such as “asthma, primary immunodeficiency” were significantly altered. In grade IV, altered GO pathways included, “negative regulation of interleukin 12 production, complement receptor-mediated signaling pathway, positive regulation of respiratory burst, T helper 2 cell differentiation, chronic inflammatory response, fever generation, T-cell proliferation involved in immune response, regulation of T helper 1 type immune response, immune response inhibiting signal transduction, and positive regulation of unsaturated fatty acid biosynthetic process” and KEGG pathways, such as “cytokine–cytokine receptor interaction, NOD-like receptor signaling pathways” were dysregulated *MR1* high gliomas. Interestingly, none of the altered GO pathways were shared among gliomas. Meanwhile, KEGG pathways commonly altered in grades II, III, and IV included allograft rejection, complement and coagulation cascades, and intestinal immune network for IGA production. Other shared pathways included graft versus host disease (III and IV), type I diabetes mellitus (III and IV), and autoimmune thyroid disease (III and IV). Taken together, alteration in these pathways results in dysregulation of processes involved in antigen processing and presentation, which impact activation and functioning of T cells. These findings represent a potential implication for impaired immunological responses in tumor recognition and clearance seen in glioma patients.

Considering this, we generated GSEA enrichment plots for GO “antigen processing and presentation” and “T-cell activation” pathways (Figure 4D). We saw a clear generalized gene enrichment for top ranking genes in each of these pathways for grades II, III, and IV, which implies that inflammatory and immune system functions are dysregulated in *MR1* high gliomas of different grades and might explain the poor OS seen in *MR1* high gliomas.

### *MR1* Expression Correlates With *MR1* Gene Promotor Methylation Status at Certain CpG Sites and Varies by Glioma Grade

**Figure 5.**
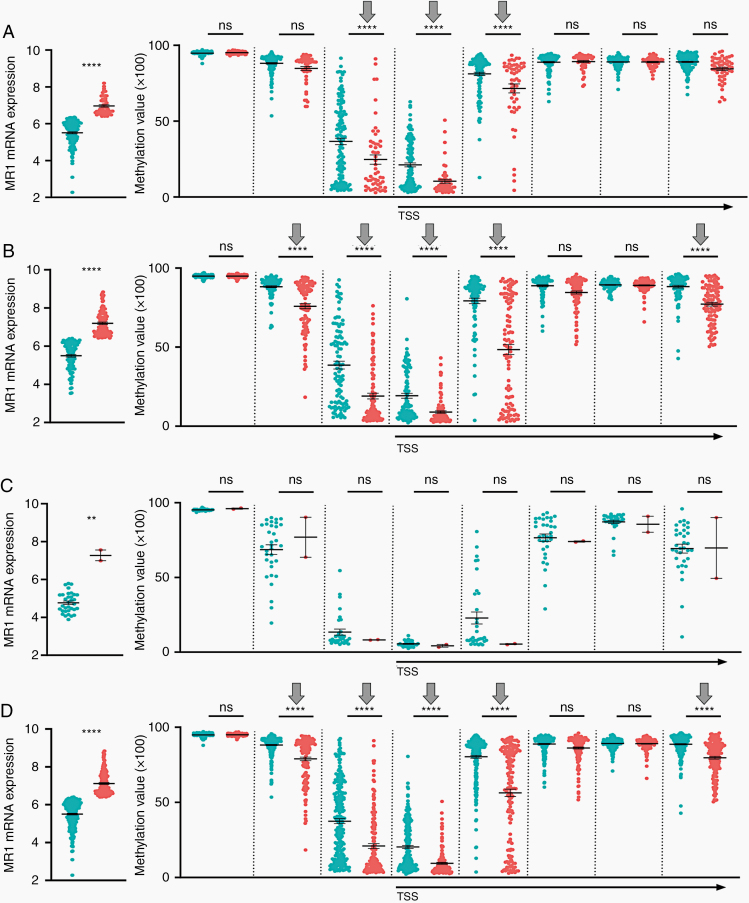
*MR1* expression correlates with *MR1* gene promoter methylation status at certain CpG sites and varies by glioma grade. The graphs on the left show distributions of low versus high *MR1*-expressing individuals in each cohort. This is followed by 8 associated CpG islands of *MR1* plotted by low versus high *MR1* expression. These plots include grades (A) II, (B) III, (C) IV, and (D) combined II/III/IV gliomas. **P < .05, ****P < .0001; ns, not significant; TSS, transcription start site.

Epigenetic modification of genes is a hallmark feature of many cancers; thus, we assessed the methylation status of *MR1* promoter specific CpG sites in all grades of *MR1* high and low gliomas. We identified 8 CpG sites within the *MR1* promotor region. There were statistically significant decreased methylation levels at 3 out of 8 sites in grade II (cg24441127, *P* < .0001; cg07025274, *P* < .0001, cg23037321, *P* < .0001; Figure 5A) and 5 out of 8 sites in grade III (cg01040850, *P* < .0001; cg24441127, *P* < .0001; cg07025274, *P* < .0001, cg23037321, *P* < .0001; and cg04903884, *P* < .0001; Figure 5B) and 0 out of 8 sites in grade IV (Figure 5C). However, when combined, all grades of glioma had 5 out of 8 CpG islands showing decreased methylation in *MR1* high-expressing glioma (cg01040850, *P* < .0001; cg24441127, *P* < .0001; cg07025274, *P* < .0001; cg23037321, *P* < .0001; and cg04903884, *P* < .0001; Figure 5D). We observed that *MR1* high-expressing grade II and III gliomas shared 3 sites that were hypomethylated between them (cg24441127, cg07025274, and cg23037321). These findings suggest that *MR1* is potentially being upregulated epigenetically in grades II and III. However, based on our data, *MR1* upregulation in grade IV is most likely not dependent on DNA methylation, but might be regulated by other mechanisms of gene expression regulation. Thus, we conclude that DNA methylation status might drive *MR1* overexpression in grades II and III, but not in grade IV. We observed that IDH-mutated (mu) gliomas in general had lower *MR1* expression levels (*P* < .0001) and a higher number of methylated CpG islands (6 out of 8 sites: cg01040850, *P* < .0001; cg24441127, *P* < .0001; cg07025274, *P* < .0001; cg23037321, cg22450342, *P* < .0001; and cg04903884, *P* < .0001) than wildtype (wt) tumors. Thus, there might a mechanistic connection between IDH mutation and *MR1* expression in primarily lower grade IDH-mutant gliomas.

**Figure 6.**
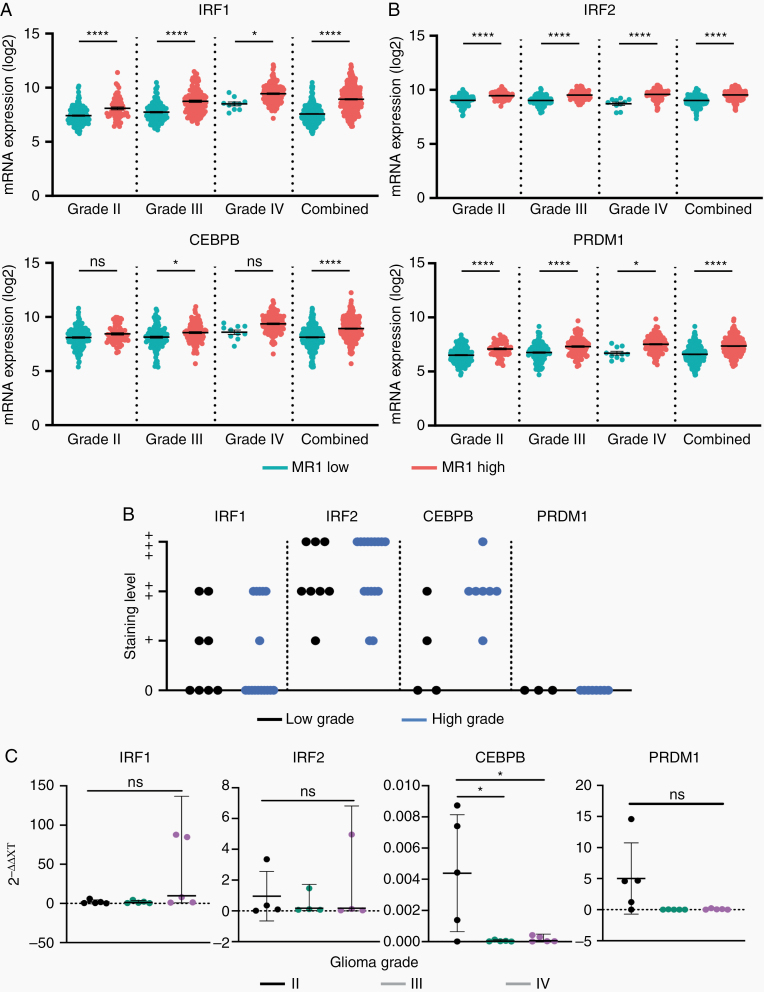
Transcription factors with binding sites on the *MR1* promoter are differentially expressed in *MR1* high versus low glioma and may regulate *MR1* gene expression. (A) The top 4 *MR1*-associated transcription factors that show significant expression differences in glioma patients (grades II, III, IV, and combined II/III/IV gliomas). (B) Compiled protein expression histology obtained from TCGA of high (III and IV) and low (II) grade gliomas. Staining levels: 0, not detected; +, low; ++, medium; +++, high. (C) qPCR analysis of transcription factors in combined grade II/III/IV gliomas. *P < .05, ***P < .001, ****P < .0001; ns, not significant.

TFs with binding sites on *MR1* promoter are differentially expressed in *MR1* high versus low glioma and may regulate *MR1* gene expression. Since TFs regulate gene expression by binding to the gene’s promoter region, we identified 26 putative TFs that were predicted to bind the *MR1* gene promoter (Supplementary Table 3). Four of these 26 TFs were found to be differentially expressed at the mRNA level in grades II, III, and IV individually and when combined. *MR1* high-expressing gliomas showed upregulation in IRF1 for grade II (*P* < .0001), III (*P* < .0001), IV (*P* < .05), and combined (*P* < .0001); IRF2 for grade II (*P* < .0001), III (*P* < .0001), IV (*P* < .0001), and combined (*P* < .0001); CEPBP for grade III (*P* < .0135) and combined (*P* < .0001); and PRDM1 for grade II (*P* < .0001), III (*P* < .0001), IV (*P* < .0235), and combined (*P* < .0001; Figure 6A). Of these, IRF1 (low grade *n* = 4, high grade *n* = 4), IRF2 (low grade *n* = 8, high grade *n* = 16), and CEBPB (low grade *n* = 2, high grade *n* = 7) also showed different and disperse levels of staining in histology (non-detected; +, low; ++, medium; +++, high) from THPA among high- and low-grade gliomas (Figure 6B; Supplementary Figure 5). Lastly, we confirmed mRNA levels of TFs from TCGA by qPCR analysis. Only CEBPB showed significant upregulation in grade II tumors (II vs III *P* < .0205; II vs IV *P* < .0236; Figure 6C), which could be explained due to the low number of samples available to us. Taken together, these results showed an upregulation of TFs that can bind to the *MR1* promoter region resulting in upregulation of *MR1* in gliomas.

## Discussion

Immune escape via defects or downregulation of MHC-I molecules is one of the main mechanisms cancer cells use to prevent elimination by cytotoxic or natural killer cells.^17^ Similarly, overexpression of nonclassical MHC-I also helps avoid targeting by cytolytic cells.^18^ MR1 is a nonclassical MHC class I-like molecule responsible for bacterial and fungal homeostasis in the mucosa. In addition to these external antigens, MR1 can activate T cells through the presentation of tumor-derived proteins.^19,20^

In the current study, we demonstrate that *MR1* expression is a predictor of OS in glioma patients. We observed that even though *MR1* mRNA levels were elevated in several solid cancers (renal, thyroid, and breast) when compared to their non-cancer controls, *MR1*’s impact on survival was glioma-specific. Among glioma grades, grade III had the highest number of days in survival difference between *MR1* high versus low tumors (difference of 1128 days). Overall, this effect of *MR1* on glioma survival was independent of IDH mutation status. A specific analysis on GBM was not possible since the majority of GBM are IDH wildtype. This data therefore indicate a critical independent link between *MR1* expression level and patient OS across all grades of glioma. We found that MR1 is primarily expressed by the glioma cells. In our GBM samples analyzed, we did not find any differences in MR1 and CD45 levels between primary or recurrent tumors; however, in most cases, higher MR1 expression is correlated with higher CD45 staining implying MR1 expression might influence immune cell infiltration. This correlation of MR1 expression by the GBM cells and immune infiltration will need to be further investigated in a mouse model experimental setting to validate correlation and understand the role of MR1 axis in the glioma microenvironment.

Within all grades of glioma, we found differential global gene expression between *MR1* high versus low. High-grade gliomas (III and IV) had a greater number of total DEGs, with grade III having the highest number of dysregulated genes and the greatest OS difference. Interestingly, we did not detect shared active or repressed DEGs among glioma grades, implying that their genetic landscapes are unique to grade. Notably, altered DEGs identified in glioma grade II are involved in several cancer processes such as epithelial–mesenchymal transition (NNMT,^21^ MFAP5^22^), cellular de-differentiation (HP),^23^ extracellular matrix degradation (SERPINA5),^24^ and cell proliferation, migration, and invasion (LTF).^25^ In grade III, altered genes are related with movement deficiency in cilia and flagella (CFAP45),^26^ neutrophil activation for immune response (PLBD1),^27^ cell cycle regulation (RASSF9),^28^ tumor-related immune response (IGKV3-11 and IGKV1-5),^29^ and tumor formation, progression, and metastasis (NTS).^30^ In grade IV, upregulated genes are linked to actin cytoskeleton regulation (STOM),^31^ inflammation and immune tolerance (IGHG4),^32^ formation and differentiation of plasma and B cells and targeting of cancer cells (CXCL13),^33^ angiogenesis and tumor invasion (MMP13),^34^ and tumorigenesis (CAPN6).^35^ This implies that MR1 can potentially impact multiple cellular processes that contribute to cancer progression, but the specific pathways impacted might be influenced by other aspects such as the aggressiveness of the glioma cells which is associated with glioma grade. However, this inference would require more detailed studies to attain a concrete conclusion.

Commonly altered pathways in high *MR1*-expressing gliomas involve antigen presentation and T-cell activation, which could influence an immunosuppressive tumor microenvironment,^13^ by altering the T-cell and NK cell function, possibly resulting in decreased tumor clearance and an ineffective antitumor immune response. On the one hand, MR1 recognition by MR1T cells was recently found to promote antitumor effects via tumor-antigen or metabolic product presentation by MR1^19,36^; however, on the other hand, anti-cytotoxic effects in tumors have been shown with other nonclassical MHC class I members by the downregulation of NK cell activity.^18,37^ The bimodal role of MR1 and T-cell interaction needs further elucidation and the role of this axis in glioma needs to be investigated.

Epigenetic events such as chromatin structure remodeling, histone modification, DNA methylation, and small noncoding RNAs can alter gene expression in the tumor microenvironment. Analysis of DNA methylation showed hypomethylation of CpG sites for the *MR1* promoter in grade II and III glioma, which can explain the upregulation of *MR1* gene expression in these groups. However, we saw no differential methylation changes in any CpG sites for grade IV. Our results suggest that *MR1* upregulation in grade IV is most likely not being modulated epigenetically by methylation status, but by other mechanisms. However, small sample size in grade IV high-expressing tumors may be a potential limitation to these findings. We hypothesize that hypomethylated CpG sites located in the *MR1* promoter are being recognized and bound by transcription activators leading to MR1 transcriptional activation. Interestingly, we saw a higher number of methylated sites in IDH mu tumors and an associated decreased expression of *MR1*, implying that IDH mu might play a role in *MR1* expression regulation. However, we found that the survival effect of *MR1* expression is independent of IDH mu status.

Gene expression is ultimately regulated by specific TF binding to the gene promoter region. We found that 4 of 26 predicted TFs (IRF1, IRF2, CEBPB, and PRDM1) were upregulated at the mRNA level in *MR1* high gliomas. Of these, IRF1, IRF2, and PRDM1 were upregulated in all grades of glioma, while CEBPB was just upregulated in grade III and combined. Notably, 3 of these TFs (IRF2, CEBPB, and PRDM1) are the main regulators of T-cell homeostasis and have been shown to hijack the optimum immune response during various diseases, including cancer.^38–43^ Some of the immunosuppressive roles described for these TFs include the promotion of exhausted and senescent CD8+ T cells during chronic viral infections by PRDM1, cancer-chemoresistance by the upregulation of regulatory T-cell recruitment through CEBPB, and the induction of T helper cell differentiation toward immunosuppressive Th2 cells by IRF2.^38–41,44,45^ Specific pathways modulated by these TFs in relation to *MR1* not yet understood.

In summary, our findings suggest that *MR1* is a prognostic marker for glioma patients and high expression is associated with poor OS. MR1 overexpression correlates with immune cell infiltration and drives diverse genetic changes that are specific to each grade and potentially promote an immune-suppressed state in glioma, enabling tumor progression. MR1 holds the valuable prognostic potential for use in glioma management and could even be therapeutically targeted. The limitation of our study is that it is primarily an in silico analysis in combination with some validation study, the mechanistic and therapeutic potential needs to be further investigated and validated using cell lines and animal models. To fully understand the mechanism by which MR1 is affecting the OS and the immune landscape functional studies need to be performed both in vitro and in vivo. In addition, to precisely target this pathway for therapeutic benefit, the significance and level of overexpression of MR1 on glioma cells as well as on possibly other immune cells need to be better characterized using preclinical models.

## Supplementary Material

vdab034_suppl_Supplementary_Materials

## Notes

**Authorship Statement.** P.K., M.B., and M.D. lead the project. P.K., M.B., M.L.V., P.L., R.P., S.S., Sh.S., R.B., and M.D. wrote the manuscript. P.K., M.L.V., S.S., I.O., P.L., R.P., S.S., Sh.S., R.B., and M.D. analyzed the data and performed genetic analysis. All authors approved the final version of the manuscript.
