# Supplementary material for: MR1 overexpression correlates with poor clinical prognosis in glioma patients
Source: Neurooncol Adv. 2021 Feb 20;3(1):vdab034. doi: 10.1093/noajnl/vdab034 (PMC8080245; doi:10.1093/noajnl/vdab034)
Supplement: vdab034_suppl_Supplementary_Materials [file vdab034_suppl_supplementary_materials.docx]

**
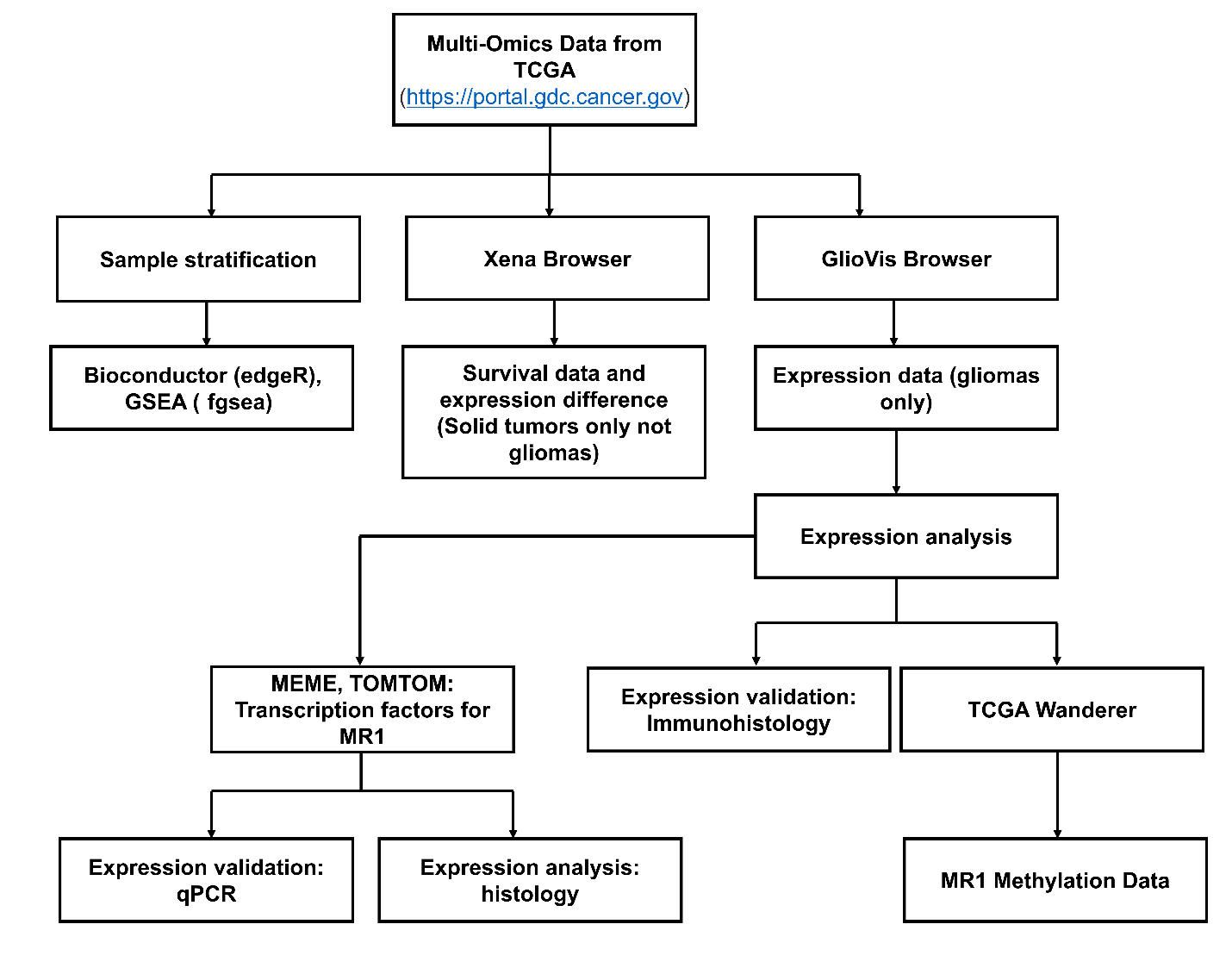
**

**Supplementary figure 1.** Schematic workflow of a multi-dimensional approach to MR1 gene analysis.


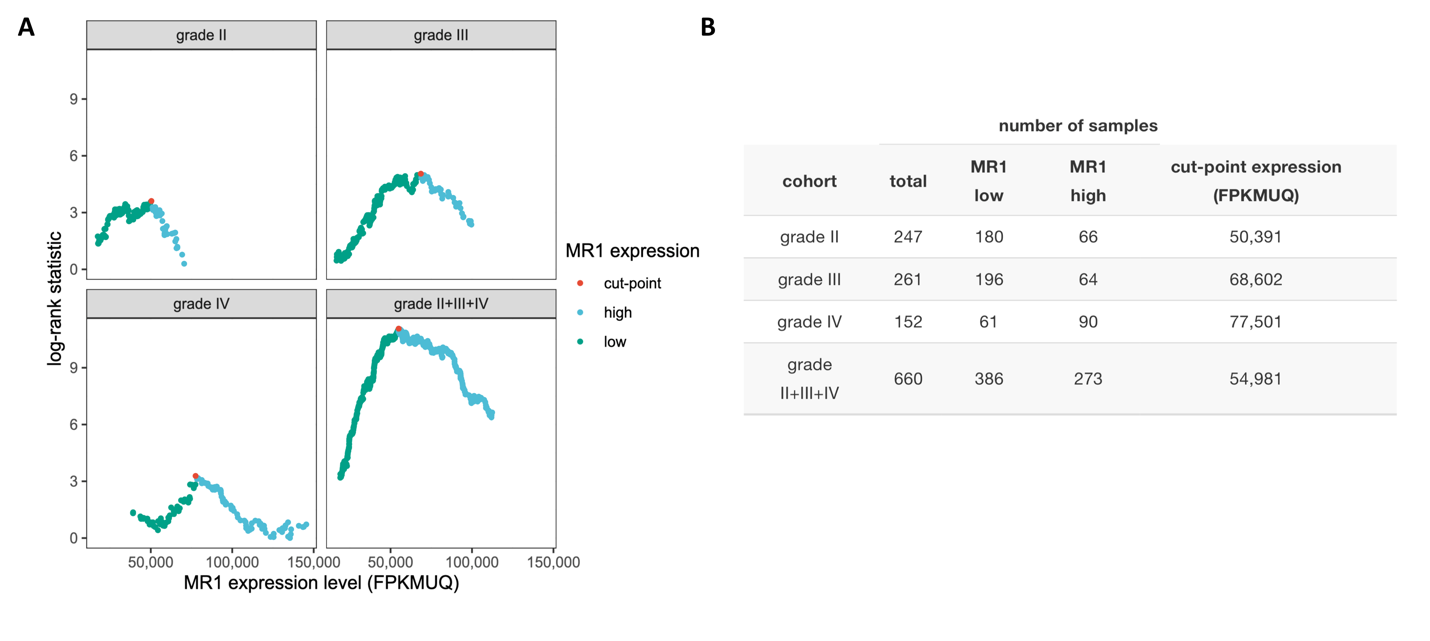


**Supplementary figure 2. MR1 expression levels in different solid malignancies.** (A) Optimal cut point of MR1 expression levels based on maximally selected rank statistics. (B) Number of TCGA primary tumor samples stratified by MR1 expression level. Samples were required to have both RNA-seq data and glioma information.


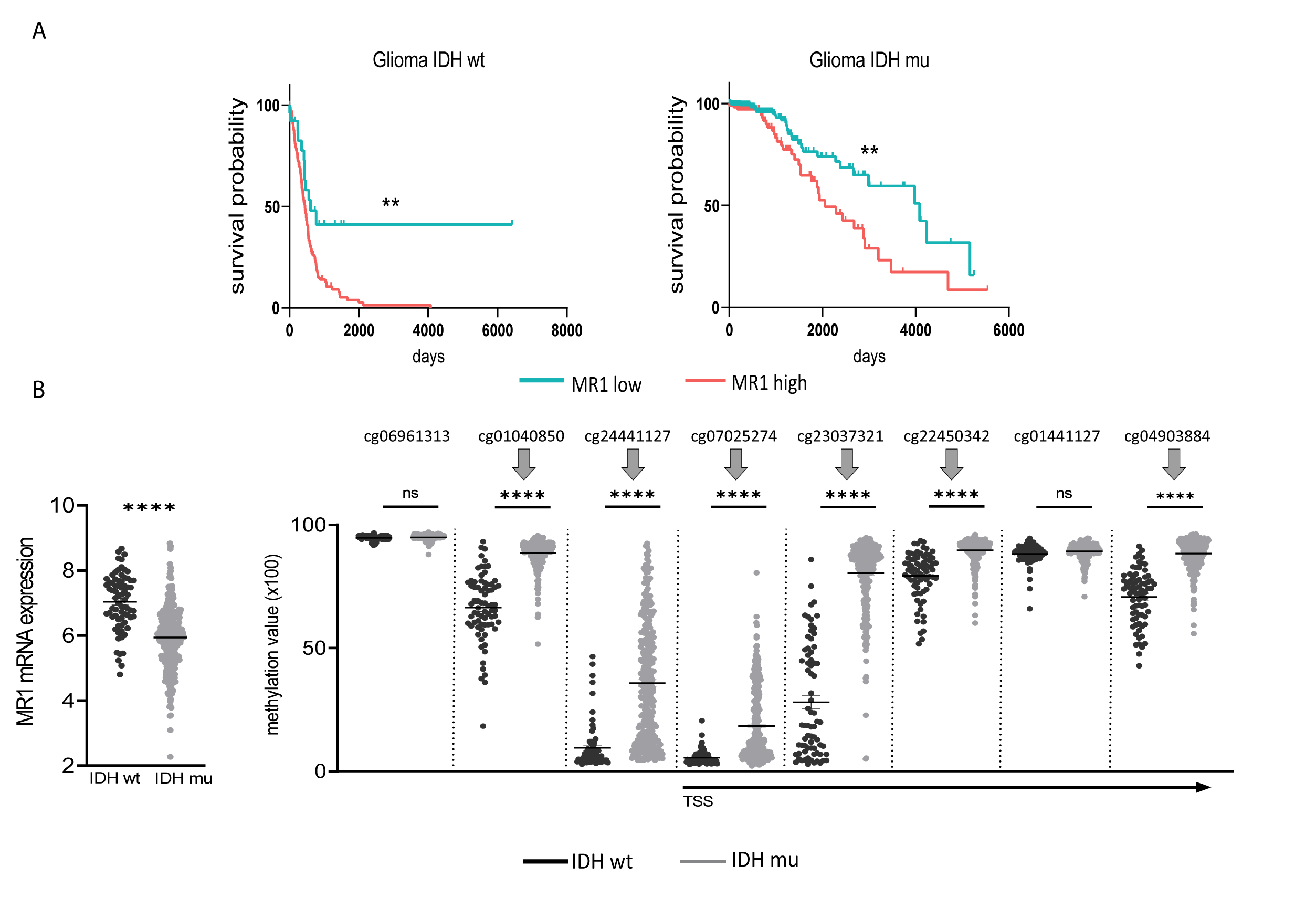


**Supplementary Figure 3.** MR1 expression levels and survival effect are independent of IDH status (wildtype or mutant) in glioma. (A) MR1 expression levels in survival are not dependent on IDH status in gliomas. (B) IDH mutant tumors have lower MR1 expression and have higher number of methylated islands of transcription factors with binding sites on MR1 promoter. *, p<0.05; **, p<0.01; ***, p<0.0001; ns, not significant.


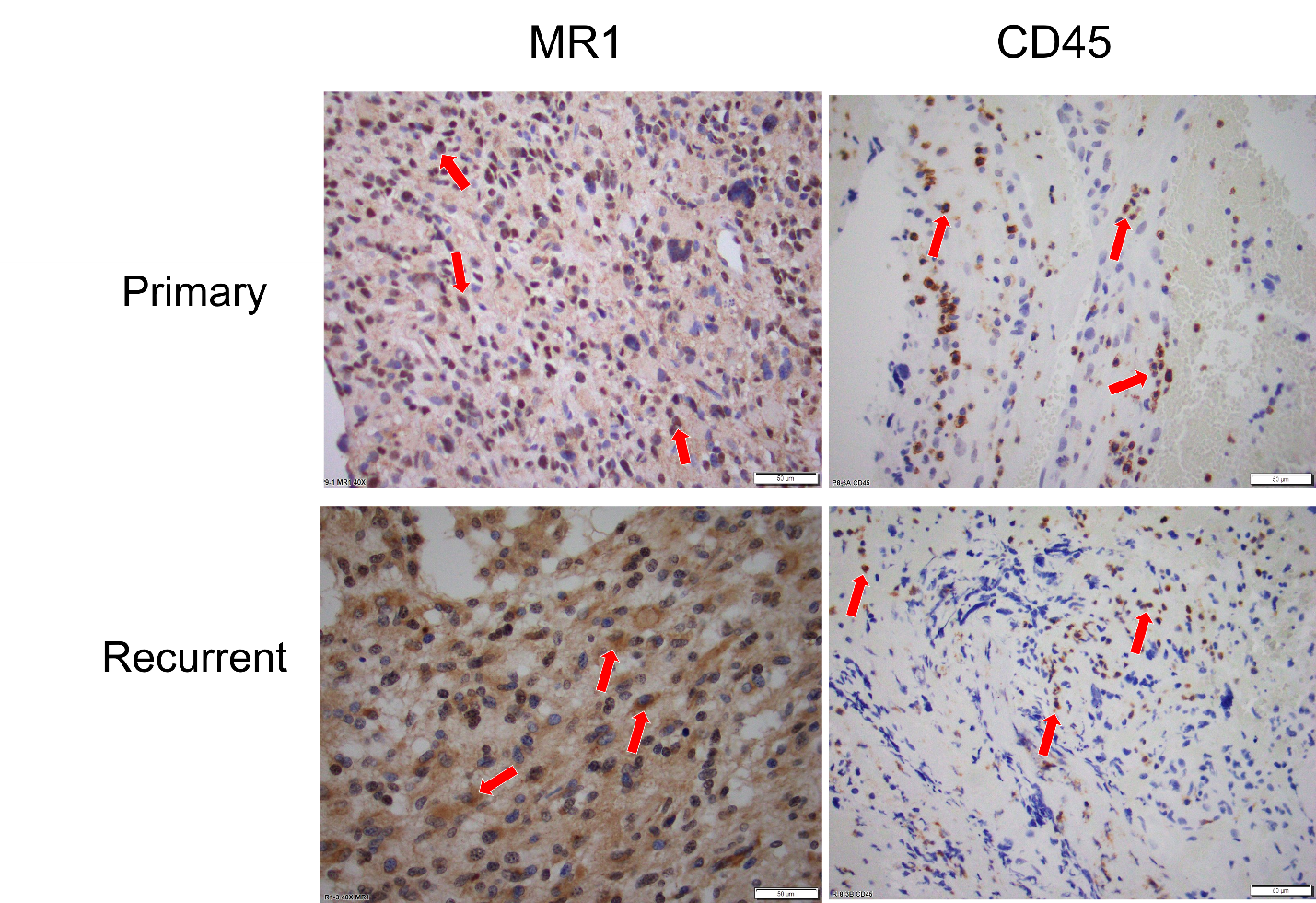


**Supplementary Figure 4.** Immunohistochemistry of MR1 and CD45 in primary and recurrent GBM tissues. MR1 expression was restricted to the cytoplasm of GBM cells in primary and recurrent tumors (pointed by arrows). CD45+ inflammatory cells were present in primary and recurrent tumors (pointed by arrows). 40x Magnification. Scale bar 50µm.

**
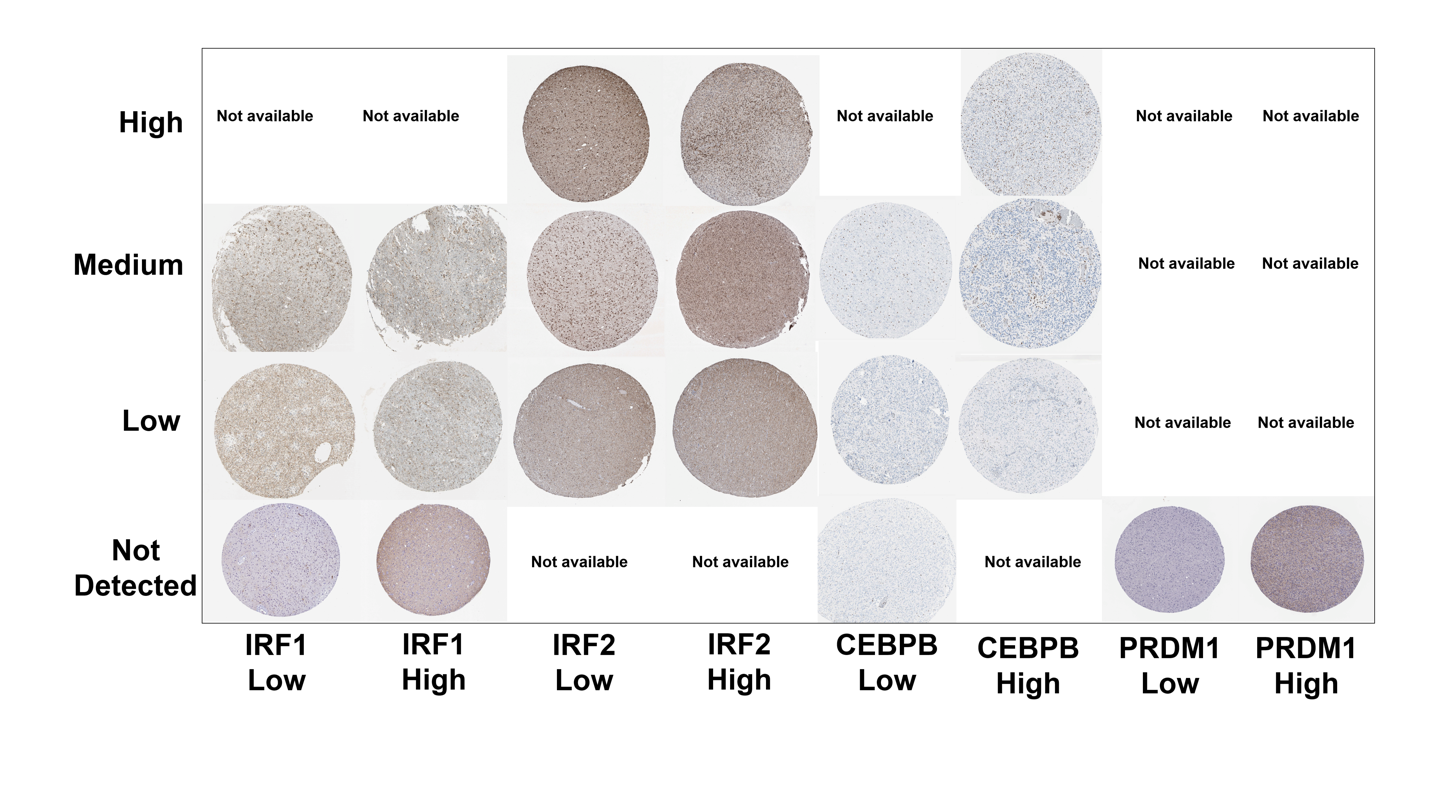
**

**Supplementary figure 5. MR1 transcription factor histology staining from The Human Protein Atlas.** Each TF has been separated to low (II) and high (III/IV) grade glioma groups. The histology is then further stratified by expression level.

**TABLES:**

**Table 1.** List of primers used for transcription factor qPCR.

| **PRIMER** | **SEQUENCE** |
| --- | --- |
| hIRF1f | agctcagctgtgcgagtgta |
| hIRF1r | tagctgctgtggtcatcagg |
| hIRF2f | atgcagaaagcgaaacgact |
| hIRF2r | ctgttgtaaggcaccggatt |
| hCEBPBf | gacaagcacagcgacgagta |
| hCEBPBr | agctgctccaccttcttctg |
| hPRDM1f | gccaagttcacccagtttgt |
| hPRDM1r | gattcgggtcagatcttcca |
| h18sf | aaacggctaccacatccaag |
| h18sr | cctccaatggatcctcgtta |

**Table 2. High MR1 expression is associated with lower survival in glioma patients.** Cox proportional-hazard ratios test demonstrating MR1 expression significantly affects survival even with common confounding variables taken into account.

|  | **Hazard Ratio** | **95% CI** | **p-value** | **Sig.** |
| --- | --- | --- | --- | --- |
| **Histology** |  |  |  |  |
| **II** | Ref. | Ref. | Ref. | Ref. |
| **II** | 1.884 | [1.198-2.962] | 0.006 | ** |
| **IV** | 2.840 | [1.553-5.194] | 0.001 | ** |
| **IDH Status** |  |  |  |  |
| **Wild-type** | Ref. | Ref. | Ref. | Ref. |
| **Mutant** | 0.317 | [1.191-0.526] | <0.001 | *** |
| **MGMT Promoter Status** |  |  |  |  |
| **Unmethylated** | Ref. | Ref. | Ref. | Ref. |
| **Methylated** | 0.288 | [0.571-1.181] | 0.288 | n.s. |
| **Age** | 1.041 | [1.027-1.055] | <0.001 | *** |
| **Gender** | 0.972 | [0.833-1.137] | 0.734 | n.s. |
| **MR1 expression** | 1.308 | [1.088-1.571] | 0.004 | ** |

**Table 3.** List of 26 transcription factors with binding site on MR1

| **Transcription factors with binding sites on MR1 promoter** |
| --- |
| 1. FOXJ2 |
| 1. IRF2 |
| 1. FOXJ3 |
| 1. LHX3 |
| 1. IRF1 |
| 1. HNF1A |
| 1. FOX04 |
| 1. AIRE |
| 1. FOXA3 |
| 1. LHX2 |
| 1. IRF8 |
| 1. CEBPB |
| 1. SRY |
| 1. FOXQ1 |
| 1. EVI1 |
| 1. NKX61 |
| 1. HNF1B |
| 1. FOXH1 |
| 1. PIT1 |
| 1. MEMCOM |
| 1. AR |
| 1. POU2F2 |
| 1. NFATC1 |
| 1. PRDM1 |
| 1. ZFP82 |
| 1. HOXA13 |

**Complementary Methods**

**Overview of sample and data collection.** We analyzed the expression patterns of MR1 in glioma and its impact on patient survival, we also investigated the DNA methylation pattern of the promotor region of MR1 gene and probed at the expression patterns of transcription factors that are predicted to bind to MR1 promoter in the glioma tissue. All expression, survival, and methylation data were obtained from The Cancer Genome Atlas (TCGA) database. The following TCGA datasets were used: GBM/LGG (glioma), THCA (thyroid), LUAD/LUSC (lung), KICH/KIRC/KIRP (renal), CESC (cervical), BRCA (breast), STAD (stomach), and SKCM (melanoma). DNA methylation (Ilumina Infinium Human Methylation450) was pulled from TCGA Wanderer (<http://maplab.imppc.org/wanderer/>), while mRNA expression (RNA-Seq) was obtained through GlioVis (<http://gliovis.bioinfo.cnio.es>) for glioma and UCSC Xena Browser (<https://xenabrowser.net>) for non-glioma solid tumors. The list of human CpG islands and their associated UCSC genes were obtained through the NCBI Gene Expression Omnibus (GEO, <https://www.ncbi.nlm.nih.gov/geo/query/acc.cgi>) with accession GPL13534. MEME and TOMTOM were used to identify transcription factor binding motifs in the MR1 promoter 16.

**Complementary Statistics**

MR1 is differentially expressed in many common solid cancers

| Tissue | mRNA levels (normal vs cancer) | P value | 95 % Confidence intervals (CI) |
| --- | --- | --- | --- |
| breast | 7.532 vs 7.788 | <0.001 | 0.1040 to 0.4082 |
| renal | 7.930 vs 8.138 | <0.0048 | 0.06372 to 0.3535 |
| glioma | 5.263 vs 6.477 | <0.0234 | 0.1645 to 2.263 |
| thyroid | 8.070 vs 8.223 | <0.0395 | 0.0074 to 0.3000 |
| cervical | 7.008 vs 7.725 | 0.2054 | -0.3950 to 1.829 |
| lung | 8.480 vs 8.238 | 0.0025 | -0.397 to -0.0854 |

MR1 and survival in solid tumors

| Tissue | OS days (MR1 low- vs high) | P value | 95 % CI |
| --- | --- | --- | --- |
| breast | 4267 vs 3941 | 0.9371 | 0.786 to1.490 |
| renal | Undefined vs 3615 | 0.1949 | Undefined |
| thyroid | Undefined | 0.8448 | Undefined |
| lung | 1338 vs 1725 | 0.3089 | 0.6366 to 0.9451 |
| melanoma | 1070 vs 857 | 0.3643 | 0.600 to 2.596 |
| stomach | 1153 vs 794 | 0.3711 | 1.061 to 1.988 |

If survival exceeds 50% at the longest time point, then median survival cannot be computed, and the median survival is reported as "undefined"

MR1 and survival in gliomas

|  | Median (days) | | P value | 95 % CI | |
| --- | --- | --- | --- | --- | --- |
| cohort | MR1 low | MR1 high |  | MR1 low | MR1 high |
| grade II | 4412 | 2875 | <0.00032 | (3571 to undefined) | (1491 to undefined) |
| grade III | 1886 | 758 | <0.0001 | (1547 to 3470) | (648 to1137) |
| grade IV | 460 | 360 | <0.0021 | (419 to 648) | (313 to 414) |
| All gliomas | 2988 | 648 | <0.0001 | (2379 to 4229) | (543 to 775) |

If survival exceeds 50% at the longest time point, then median survival cannot be computed, and the median survival is reported as "undefined"

MR1 expression and methylation status at CpG sites with binding affinity for the MR1 promoter

| CpG sites | Grade II (p value, 95% CI) | Grade III (p value, 95% CI) | Grade IV (p value, 95% CI) | All grades combined (p value, 95% CI) |
| --- | --- | --- | --- | --- |
| cg06961313 | >0.9999,  -6.931 to 6.302 | >0.9999,  -6.887 to 6.901 | >0.9999,  -35.83 to 34.22 | >0.9999,  -4.917 to 4.649 |
| cg01040850 | 0.9332,  -3.244 to 9.989 | <0.0001,  5.437 to 19.23 | >0.9999,  -43.31 to 26.74 | <0.0001,  4.468 to 14.03 |
| cg24441127 | <0.0001,  5.335 to 18.57 | <0.0001,  12.69 to 26.48 | >0.9999,  -29.82 to 40.23 | <0.0001,  11.74 to 21.30 |
| cg07025274 | <0.0001,  4.155 to 17.39 | <0.0001,  3.474 to 17.26 | >0.9999,  -33.68 to 36.36 | <0.0001,  6.151 to 15.72 |
| cg23037321 | <0.0001,  3.004 to 16.24 | <0.0001,  23.87 to 37.66 | 0.9379,  -17.54 to 52.51 | <0.0001,  19.20 to 28.76 |
| cg22450342 | >0.9999,  -6.968 to 6.265 | 0.7582,  -2.654 to 11.14 | >0.9999,  -32.51 to 37.53 | 0.8632,  -2.094 to 7.472 |
| cg01441127 | >0.9999,  -6.553 to 6.680 | >0.9999,  -6.611 to 7.178 | >0.9999,  -33.45 to 36.60 | >0.9999,  -4.647 to 4.919 |
| cg04903884 | 0.5450,  -1.982 to 11.25 | <0.0001,  4.173 to 17.96 | >0.9999,  -35.47 to 34.57 | <0.0001,  4.268 to 13.83 |

IDH status and MR1 expression levels effects on methylation of CpG islands with binding affinity for the MR1 promoter

| CpG sites | IDH mutant vs. wildtype (p value, 95%CI ) |
| --- | --- |
| cg06961313 | >0.9999, -5.330 to 4.965 |
| cg01040850 | <0.0001, -27.26 to -16.96 |
| cg24441127 | <0.0001, -31.43 to -21.13 |
| cg07025274 | <0.0001, -17.94 to -7.647 |
| cg23037321 | <0.0001, -57.62 to -47.32 |
| cg22450342 | <0.0001, -15.55 to -5.252 |
| cg01441127 | >0.9999, -6.256 to 4.038 |
| cg04903884 | <0.0001, -22.79 to -12.49 |

Transcription factors and MR1 expression levels in gliomas

| TF | Groups | P value | 95% CI |
| --- | --- | --- | --- |
| IRF1 | MR1 Low GII vs. MR1 High GII | <0.0001 | -1.103 to -0.2388 |
|  | MR1 Low GIII vs. MR1 High GIII | <0.0001 | -1.376 to -0.6399 |
|  | MR1 Low GIV vs. MR1 High GIV | 0.0479 | -1.881 to -0.004528 |
|  | MR1 Low Combined vs. MR1 High Combined | <0.0001 | -1.577 to -1.133 |

| IRF2 | MR1 Low GII vs. MR1 High GII | <0.0001 | -0.6192 to -0.2458 |
| --- | --- | --- | --- |
|  | MR1 Low GIII vs. MR1 High GIII | <0.0001 | -0.6549 to -0.3368 |
|  | MR1 Low GIV vs. MR1 High GIV | <0.0001 | -1.210 to -0.4906 |
|  | MR1 Low Combined vs. MR1 High Combined | <0.0001 | -0.6123 to -0.4206 |

| CEBPB | MR1 Low GII vs. MR1 High GII | -0.3384 | -0.7671 to 0.09030 |
| --- | --- | --- | --- |
|  | MR1 Low GIII vs. MR1 High GIII | -0.4148 | -0.7800 to -0.04961 |
|  | MR1 Low GIV vs. MR1 High GIV | -0.7804 | -1.712 to 0.1512 |
|  | MR1 Low Combined vs. MR1 High Combined | -0.8004 | -1.020 to -0.5803 |

| PRDM1 | MR1 Low GII vs. MR1 High GII | <0.0001 | -0.9180 to -0.2149 |
| --- | --- | --- | --- |
|  | MR1 Low GIII vs. MR1 High GIII | <0.0001 | -0.8504 to -0.2515 |
|  | MR1 Low GIV vs. MR1 High GIV | 0.0235 | -1.590 to -0.06182 |
|  | MR1 Low Combined vs. MR1 High Combined | <0.0001 | -0.9397 to -0.5788 |
